# Synthetic approaches for advanced multi-block anion exchange membranes†

**DOI:** 10.1039/c9ra03888d

**Published:** 2019-07-05

**Authors:** Dongwon Shin, Adam F. Nugraha, Farid Wijaya, Sojeong Lee, Eunyoung Kim, Jieun Choi, Hyoung-Juhn Kim, Byungchan Bae

**Affiliations:** Fuel Cell Laboratory, Korea Institute of Energy Research Daejeon 34129 Republic of Korea bcbae@kier.re.kr; Department of Renewable Energy Engineering, University of Science and Technology Daejeon 34113 Republic of Korea; Fuel Cell Research Center, Korea Institute of Science and Technology Seoul 02792 Republic of Korea

## Abstract

Despite our ability to post-functionalize poly(arylene ether sulfone) multi-block copolymers by rapid chloromethylation, bromination, or acylation, with degrees of functionalization that exceeded 70% in a few hours, materials formed during attempts to prepare fully post-functionalized multi-block copolymers are poorly soluble due to undesired side reactions, such as crosslinking or di-bromination. In particular, clustered reactive sites in multi-block copolymers increase the chance of self-reactions between polymer backbones, resulting in the formation of by-products. On the other hand, the authentic multi-block copolymer with good solubility and high molecular weight was successfully synthesized using functionalized monomers. Despite its low ion-exchange capacity, the resulting multi-block copolymer outperformed the commercial FAA-3-30 membrane in terms of anion conductivity, even under low relative humidity conditions.

## Abstract 2

Multi-block copolymers bearing ionic functional groups were synthesized through direct polymerization or post-functionalization.

## Graphical abstract

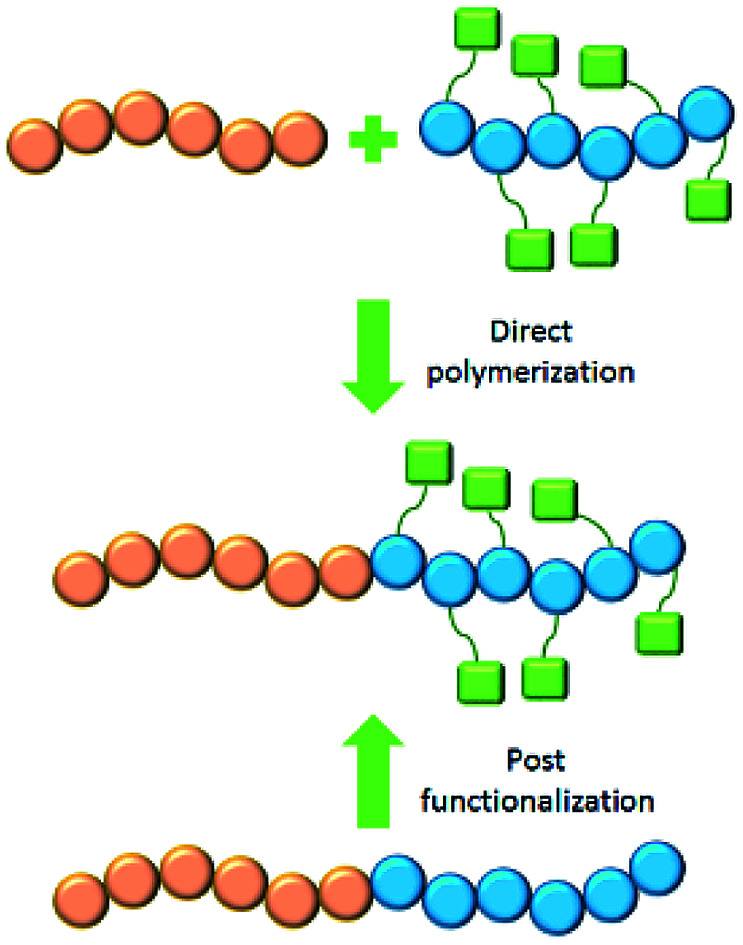

## Introduction

Anion exchange membranes (AEMs) are composed of cationic functional groups attached to polymer matrices. They selectively transport anions while blocking neutral species and cations. AEMs have been used in a variety of applications such as water treatment (*e.g.*, electrodialysis, and membrane capacitive deionization) and energy devices (*e.g.*, redox flow batteries, fuel cells, and reverse electrodialysis) due to their good permselectivities.^1–4^ Among various applications, redox flow batteries and fuel cells are representative, since these energy devices provide promising solutions that overcome global climate change issues and fluctuations in the grid from renewable energy sources.^5–8^ The main advantage of using an AEM in a redox flow battery is the barrier properties of vanadium ions that result in electrostatic repulsion. Similarly, the AEM prevents leakage of the liquid electrolyte in a conventional alkaline fuel cell and corrosion by the highly alkaline electrolyte, as examples.^9,10^ For these reasons, AEMs are key components in energy devices. However, poor electrochemical performance and the long-term durabilities of AEMs remain obstacles for commercialization.^7,11,12^ Therefore, the most significant challenge is the development of advanced AEMs that exhibit high anion conductivities, and chemical and mechanical stabilities.

To date, a variety of AEMs based on aromatic polymers, such as poly(arylene ether sulfone)s, poly(arylene ether ketone)s, polybenzimidazoles, polyphenylenes, and poly(phenylene oxide)s, have been developed because of their good thermal and mechanical stabilities, and ease of synthesis and functionalization.^13–20^ However, these materials are random copolymers, and the random architectures of their rigid aromatic groups require high ion exchange capacities (IECs) to achieve useful ion conductivities. In addition, anion (OH^−^) conductivity is intrinsically lower than proton (H^+^) conductivity due higher proton mobility.^21^ With this background in mind, multi-block copolymers are candidates that should improve the electrochemical performance of AEMs because their well-defined hydrophilic–hydrophobic phase-separated morphologies enhance their conductivities in a similar manner to proton exchange membranes (PEMs).^22^ Furthermore, highly dense hydrophobic blocks support the mechanical properties of AEMs, particularly when the membrane is partially hydrated or swollen in water.^23^ However, multi-block copolymers with high molecular weights are difficult to synthesize because of the need for additional coupling reactions between hydrophilic and hydrophobic oligomers that are known to have lower telechelic end-group reactivities than monomers involved in random copolymerization, due to their larger molecular sizes. For these reasons, most AEM studies involve random copolymers.^19,24,25^

Recently, the chloromethylations and brominations of aromatic random copolymers have been widely studied for the introduction of cationic moieties following the quaternization (Menshutkin reaction) of benzylic halides with tertiary amines.^17,26^ Although chloromethylation requires toxic chemicals such as chlorinated solvents and chloromethyl methyl ether (CMME), it is a very simple and fast reaction. In a similar manner, radical bromination is also easily performed by thermal initiation over short reaction times.^17^ In addition, both chloromethylation and bromination are cost-beneficial because expensive metal catalysts and complicated purification steps are not required; these reactions are also selective and are controlled by the electron densities of the aromatic rings. Therefore, post-functionalization through chloromethylation or bromination appears to be a good approach for incorporating functional groups into multi-block copolymers, since the block-copolymer architecture is retained following functionalization.

It is very important that chloromethylation and bromination reaction conditions are optimized in order to obtain a concentration of anion exchange functional groups that matches the targeted chemical structure, while avoiding side reactions. In particular, precise conditions are necessary in order to functionalize a multi-block copolymer, since the phase-separation advantages of the hydrophilic and hydrophobic blocks in a multi-block copolymer are not always preserved when small changes are made to its chemical structure. However, to the best of our knowledge, no studies aimed at optimizing the functionalization conditions for multi-block copolymers have been reported.

Herein, we report optimized reaction conditions for the post-functionalization of poly(arylene ether sulfone)-based multi-block copolymers through chloromethylation or bromination. Multi-block copolymer precursors were designed such that the hydrophilic blocks in the corresponding copolymers are exclusively functionalizable to give the desired multi-block architectures. The reaction conditions were controlled by changing the amount of catalyst (or initiator), reagents (CMME or *N*-bromosuccinimide), reaction times, and temperatures. In addition, in order to promote chloromethylation and bromination, different Lewis-acid catalysts and thermal initiators were explored, respectively. A poly(arylene ether sulfone)-based random copolymer was also post-functionalized in order to compare its reactivity toward functionalization with those of the multi-block copolymers. Changes in chemical structure and the degree of functionalization before and after reaction were examined by ^1^H NMR spectroscopy. Although the reactions were complete within a few hours, the multi-block copolymers were poorly soluble when the degree of functionalization exceeded a specific level, whereas fully functionalized random copolymers exhibited no solubility issues. These observations are ascribable to side reactions that occur during functionalization. Consequently, we also provide an alternative method for the synthesis of multi-block copolymers bearing anion exchange groups that involves a functionalized monomer.

## Experimental

### Materials

ZnCl_2_, SnCl_4_, CMME, benzoyl peroxide (BPO), azobisisobutyronitrile (AIBN), *N*-bromosuccinimide (NBS), 1,1,2,2-tetrachloroethane (TCE), CDCl_3_, polysulfone (PSU) random copolymer (average *M*_n_ ∼ 22 000), bis(4-fluorophenyl) sulfone (DFDPS), 4,4′-(hexafluoroisopropylidene)diphenol (6F-BPA), 4,4′-dihydroxybiphenyl (BP), methyl iodide, Cs_2_CO_3_, K_2_CO_3_, CaCO_3_, deuterated chloroform (CDCl_3_) and *N*,*N*-dimethylsulfoxide (DMSO-*d*_6_), 1-methyl-2-pyrrolidinone (NMP), and *N*,*N*-dimethylacetamide (DMAc) were purchased from Sigma-Aldrich. Sodium chloride (NaCl), methanol and hydrochloric acid were supplied by Samchun Chemicals and used as received. Tetra(trimethylaminemethylene)-4,4′-dihydroxydiphenylether (TADHDPE) was synthesized following our previous report.^27^ The FAA-3-30 membrane was purchased from FUMATECH as a reference AEM, and prior to its use, the counter ion was converted to chloride anion using aqueous 1 M NaCl solution.

### Synthesis of poly(arylene ether sulfone) multi-block copolymers (PAESs)

As precursors for chloromethylation and bromination, PAESs were synthesized by procedures provided in the ESI.†

### Chloromethylation

The following is a general procedure for the chloromethylation of a PAES. The PAES was dissolved in TCE (2.5% w/v) in a two-neck flask under nitrogen. ZnCl_2_ or SnCl_4_ (1–2 molar equiv. with respect to the biphenyl groups in the polymer) was added as the Lewis-acid catalyst, followed by the addition of CMME (20–80 molar equiv. with respect to the biphenyl groups in the polymer). The reaction time and temperature were also controlled in order to optimize the degree of chloromethylation (DC, 100% means the presence of two benzyl chloride groups on the biphenyl unit); they ranged from 2 to 24 h and from 35 to 50 °C, respectively. The reaction mixture was cooled and poured into methanol to obtain the product, which was collected by filtration, washed several times with methanol, and dried at 80 °C under vacuum. For comparison, PSU was chloromethylated using a similar procedure.

### Bromination

PAES was dissolved in TCE (7.5% w/v) in a two-neck flask, under nitrogen. BPO or AIBN (0.02–0.10 equiv. with respect to the active sites of the polymer) and NBS (0.5–2.0 equiv. with respect to the active sites of the polymer) were added as initiator and reagent, respectively. The reaction time and temperature were adjusted within the 1–12 h and 65–85 °C ranges, respectively, in order to maximize the degree of bromination (DB) and minimize the degree of di-bromination (DBB). After cooling to room temperature, the reaction mixture was poured into methanol to terminate the reaction. The product was collected by filtration and dried at 80 °C under vacuum.

### Synthesis of the tetra-trimethylbenzylammonium functionalized multi-block copolymer

The hydrophilic oligomer was prepared in the following way. A round-bottom flask equipped with a condenser and nitrogen inlet/outlet valves was charged with DFDPS (1.32 mmol, 0.34 g) and TADHDPE (1.16 mmol, 0.50 g). After dissolving the monomers in 5 mL of DMAc at 100 °C, CaCO_3_ (11.61 mmol, 1.16 g) was added to the solution to prevent ether–ether exchange reactions, and CsCO_3_ (2.32 mmol, 0.83 g) was used as the catalyst. The solution was precipitated into 1 M aqueous HCl. The white powder was collected by filtration, washed with methanol several times, and dried overnight under vacuum at 80 °C.

The hydrophobic block was synthesized in a similar manner to the hydrophilic oligomer. 6F-BPA (5.94 mmol, 2.00 g) and DFDPS (6.34 mmol, 1.61 g) were dissolved in 8 mL of DMAc at 140 °C. K_2_CO_3_ (8.90 mmol, 1.23 g) was added as the catalyst. Oligomerization was carried out at 140 °C for 4 h. Then, the oligomer was end-capped with BP (5.70 mmol, 1.06 g). The solution was precipitated into water, the product was collected by filtration, washed several times with methanol, and dried under vacuum at 80 °C.

Polymerization was carried out in the following manner. The hydrophilic oligomer (0.03 mmol, 0.34 g) and the hydrophobic oligomer (0.03 mmol, 0.18 g) were dissolved in NMP at 120 °C. CaCO_3_ (3.40 mmol, 0.34 g) and K_2_CO_3_ (0.12 mmol, 0.02 g) were added and the mixture was stirred at 120 °C for 24 h. The polymer was collected by filtration following precipitation of the solution into 1 M aqueous HCl, washed with methanol, and dried under vacuum at 80 °C.

The polymer was dissolved in DMAc and was quaternized with excess methyl iodide (100 equiv. with respect to tertiary amine groups) at room temperature for 48 h. The final product (B-Q-1) was obtained after washing with water and drying under vacuum at 80 °C. The membrane was cast on a glass plate with 30 μm thickness, and the counter ion was converted to the chloride anion using aqueous 1 M NaCl solution.

### Characterization

^1^H NMR spectra were acquired on an AVANCE III 600 NMR spectrometer in CDCl_3_ or DMSO-*d*_6_, with chemical shifts referenced to tetramethylsilane. DC, DB, DBB, and IEC values were calculated from integrated peaks in the ^1^H NMR spectra.

The molecular weights of oligomers and polymers were monitored by gel-permeation chromatography (GPC) using a Younglin YL 9112 isocratic pump, a YL 9120 UV-visible detector (Younglin Instruments, Korea), and a KF-805L column. DMAc containing 0.05 M LiBr was used as the eluent. All samples were filtered through a 0.2 μm polytetrafluoroethylene (PTFE) syringe filter to remove insoluble components, and polystyrene was used as the reference.

Water uptake was calculated on the basis of a weight ratio of the absorbed water to the dry membrane. The dry membrane weight was obtained after drying at 120 °C under vacuum for 24 h, and the wet membrane weight was measured after immersing in deionized water at room temperature for 24 h. Water vapor sorption experiment was conducted using TGA Q5000SA (TA Instruments) at RH ranging from 30 to 70% at 70 °C.

Chloride anion conductivity was measured by electrochemical impedance spectroscopy by using a membrane conductivity cell (WonATech). The temperature and humidity were controlled using a fuel cell test station (CNL).

## Results and discussion

### Chloromethylation

**Scheme 1:**
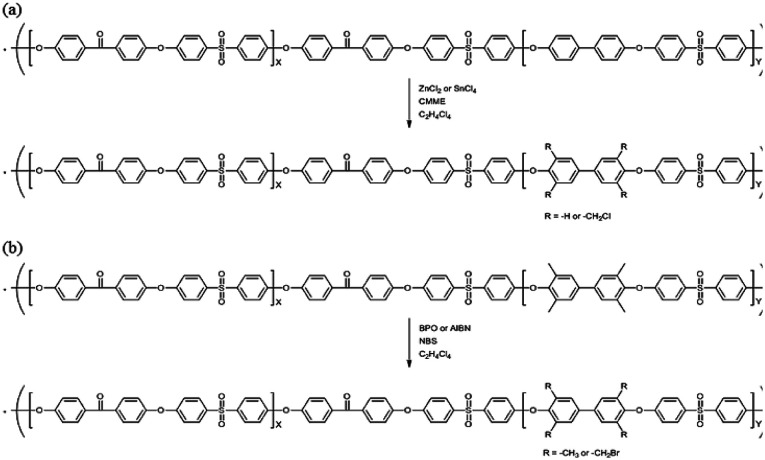
Functionalization of a poly(arylene ether sulfone) multi-block copolymer by (a) chloromethylation and (b) bromination.

**Fig. 1:**
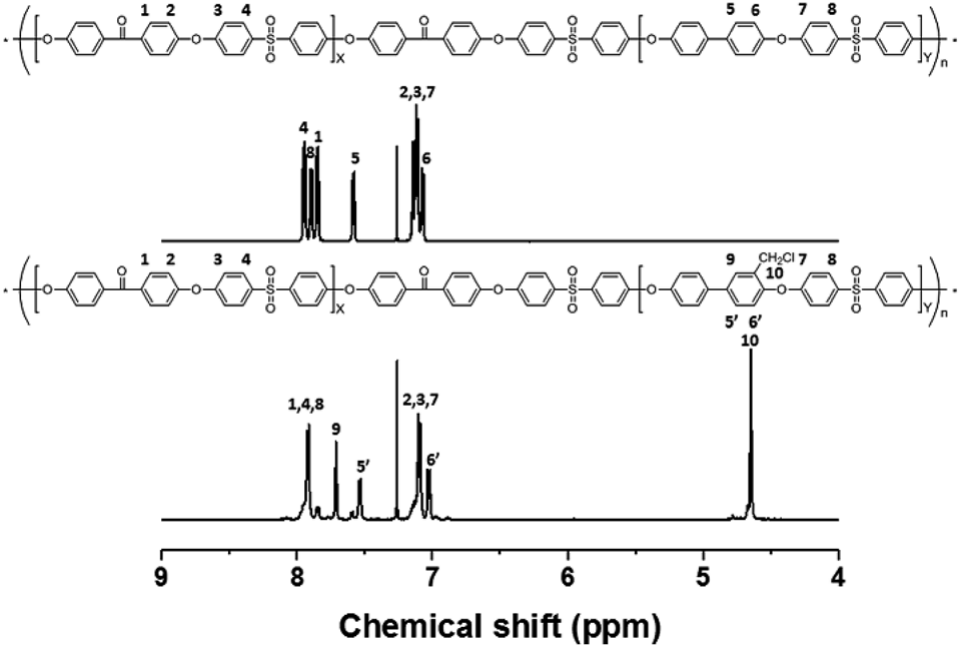
^1^H NMR spectra of a poly(arylene ether sulfone) multi-block copolymer before and after chloromethylation.

**Scheme 2:**
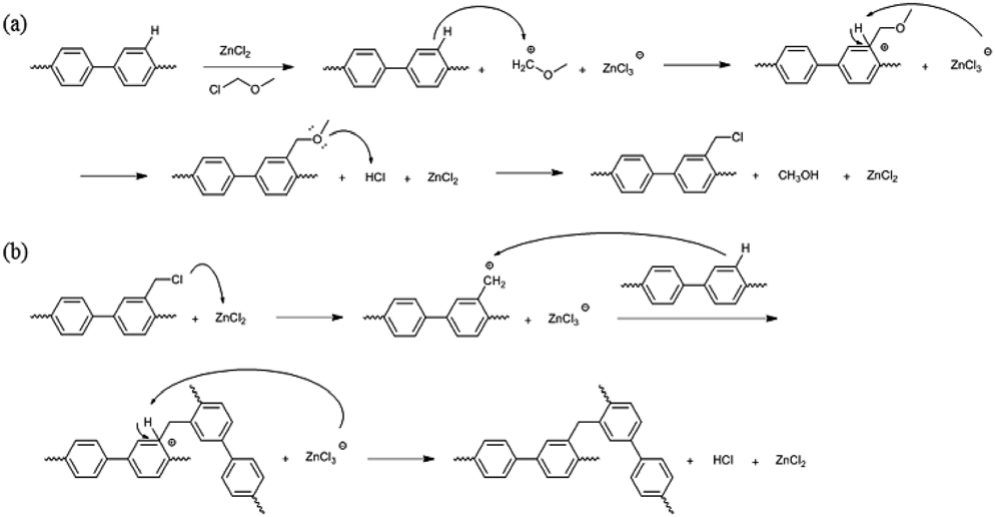
Potential mechanisms for (a) chloromethylation and (b) crosslinking.

The precursor to the PAES multi-block copolymer was synthesized from a ketone-containing hydrophobic oligomer and a biphenyl-containing hydrophilic oligomer; these units were characterized by ^1^H NMR spectroscopy and GPC (Scheme S1, Fig. S1 and S2†). We prepared a series of chloromethylated PAES-based polymers following the procedure shown in Scheme 1a; they are referred to as “B–C-*x*”, where B and C indicate “block copolymer” and “chloromethylation”, respectively, while *x* refers to the batch number. Chloromethyl groups were selectively introduced onto the biphenyl units targeted as hydrophilic blocks, since the electron-withdrawing groups (*e.g.*, ketone and sulfone) in the hydrophobic blocks deactivate their nucleophilicities. ^1^H NMR spectroscopy was used to identify chemical structures following chloromethylation, and to determine the DC. Fig. 1 shows representative ^1^H NMR spectra of a PAES multi-block copolymer before and after chloromethylation. A signal appeared at 4.6 ppm in the spectrum of chloromethylated PAES, which is ascribable to the formation of chloromethyl groups. In addition, peaks corresponding to protons 5 and 6 (see Fig. 1) were slightly shifted following chloromethylation, which is indicative of the selective introduction of chloromethyl moieties onto the biphenyl units, as described in Scheme 2a. However, unreacted biphenyl groups still remain (*e.g.*, proton 5 at 7.6 ppm and proton 8 at 7.8 ppm, red arrows in Fig. 1). The DC was determined from the relative (integrated) intensities of the chloromethyl (protons 10) peak and those of protons in the main polymer chain (protons 1, 4, and 8); the DC was found to be 80%.

**Fig. 2:**
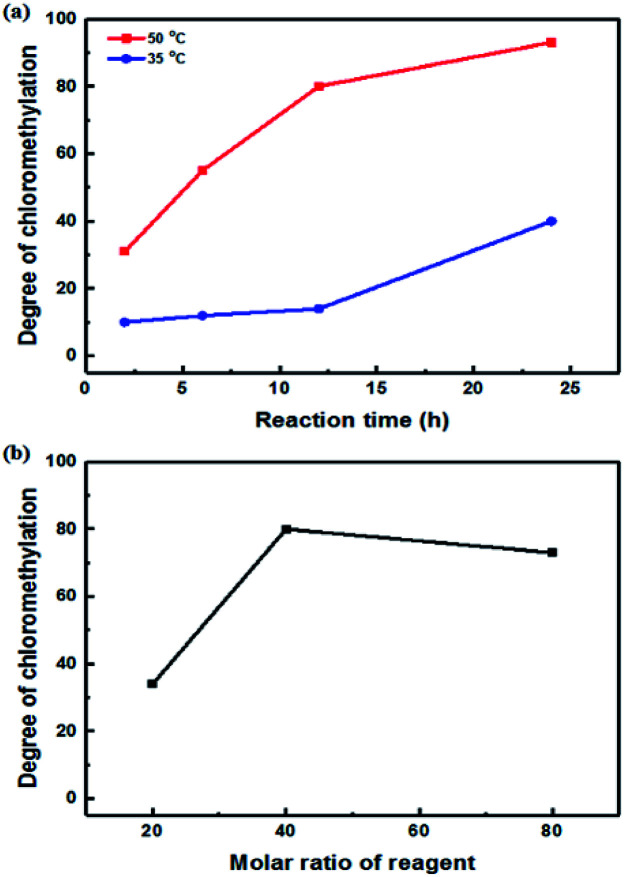
Degree of chloromethylation as a function of (a) reaction time and (b) the molar ratio of reagent.

**Fig. 3:**
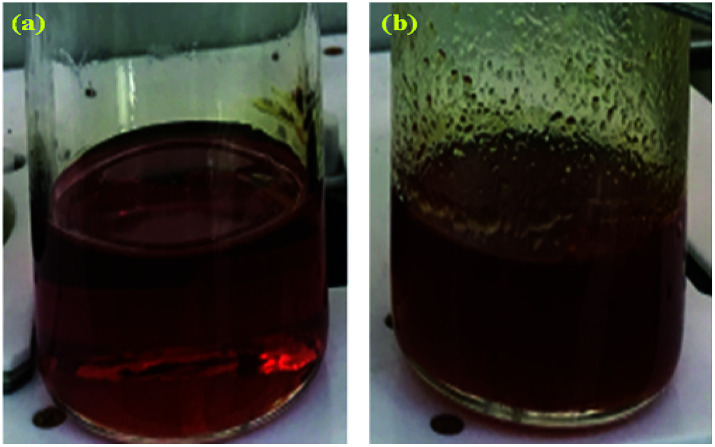
Photographic images of the reaction solution after chloromethylation (a) without and (b) with crosslinking.

A high degree of functionalization is essential to maintain the chemical structure of the designed block copolymer because a low degree of functionalization decreases the hydrophilicities of the hydrophilic blocks resulting a low degree of phase separation between the hydrophobic and hydrophilic blocks. With the aim of optimizing the reaction condition, chloromethylation was carried out under a variety of reaction conditions by altering the amounts of catalyst and CMME, as well as the reaction time and temperature, the results of which are summarized in Table 1. The reaction time was increased from 2 to 24 h to investigate the effect of time on the DC. We selected ZnCl_2_ and CMME as the Lewis-acid catalyst and reagent, respectively. The reaction proceeded slowly to 12 h; further reaction provided a DC of 40% after 24 h at 35 °C. Chloromethylation is known to be a very fast reaction that is usually complete in a few hours. However, in our case, the reaction with 1 equiv. of catalyst at 35 °C was insufficient for effective chloromethylation; hence the amount of catalyst was increased to 2 equiv. and the reaction was performed at 50 °C; this reaction was faster than that at 35 °C and gave a relatively high DC of 80% after 12 h, as shown in Fig. 2a. Although the DC was 93% after 24 h, the product was partially insoluble owing to crosslinking, as shown in Scheme 2b and Fig. 3; crosslinking was also clearly evidenced by ^1^H NMR spectroscopy (Fig. S3†). We also varied the amount of reagent from 20 to 80 equiv. relative to the active sites (biphenyl units) on the polymer backbone in order to obtain a DC of 100% and to suppress crosslinking. We postulated that the higher concentration of CMME would increase the rate of desirable chloromethylation during the initial stages of reaction; in other words, the remaining biphenyl groups were expected to be consumed quickly at the start of the reaction, which would decrease the probability of the crosslinking reaction shown in Scheme 2. The DC was much lower with 20 equiv. of CMME than with 40 equiv., whereas a DC of 73% was obtained with 80 equiv. of CMME after a 2 h reaction, as illustrated in Fig. 2b. Nevertheless, the reaction formed a gel with a few hours with 80 equiv. of CMME, and was hard to control. The use of excess amounts of catalyst and reagent are very harsh conditions that appear to increase the rate of crosslinking as well as the desired chloromethylation. On the basis of these results, we conclude that 2 equiv. of ZnCl_2_ and 40 equiv. of CMME at 50 °C for 12 h are optimal for the chloromethylation of a PAES multi-block copolymer.

**Table 1:** Summary of the chloromethylation reaction conditions

Sample	Catalyst^a^ (equiv.)	Reagent^b^ (equiv.)	Temperature (°C)	Time (h)	DC (%)
B–C-1	1	40	35	2	10
B–C-2	1	40	35	6	12
B–C-3	1	40	35	12	14
B–C-4	1	40	35	24	40
B–C-5	2	40	50	2	31
B–C-6	2	40	50	6	55
B–C-7	2	40	50	12	80
B–C-8	2	40	50	24	93^c^
B–C-9	2	20	50	12	34
B–C-10	2	80	50	2	73
B–C-11	2	40	50	2	—d
B–C-12	2	40	50	6	—d
B–C-13	2	40	50	12	—d
B–C-14	2	40	50	24	—d
R–C-1	2	40	50	2	100

a Molar ratio of catalyst to active sites (B–C-1 to B–C-10 and R–C-1, catalyst = ZnCl_2_; B–C-11 to B–C-14, catalyst = SnCl_4_).

b Molar ratio of reagent to active sites.

c Partially insoluble.

d Insoluble.

**Fig. 4:**
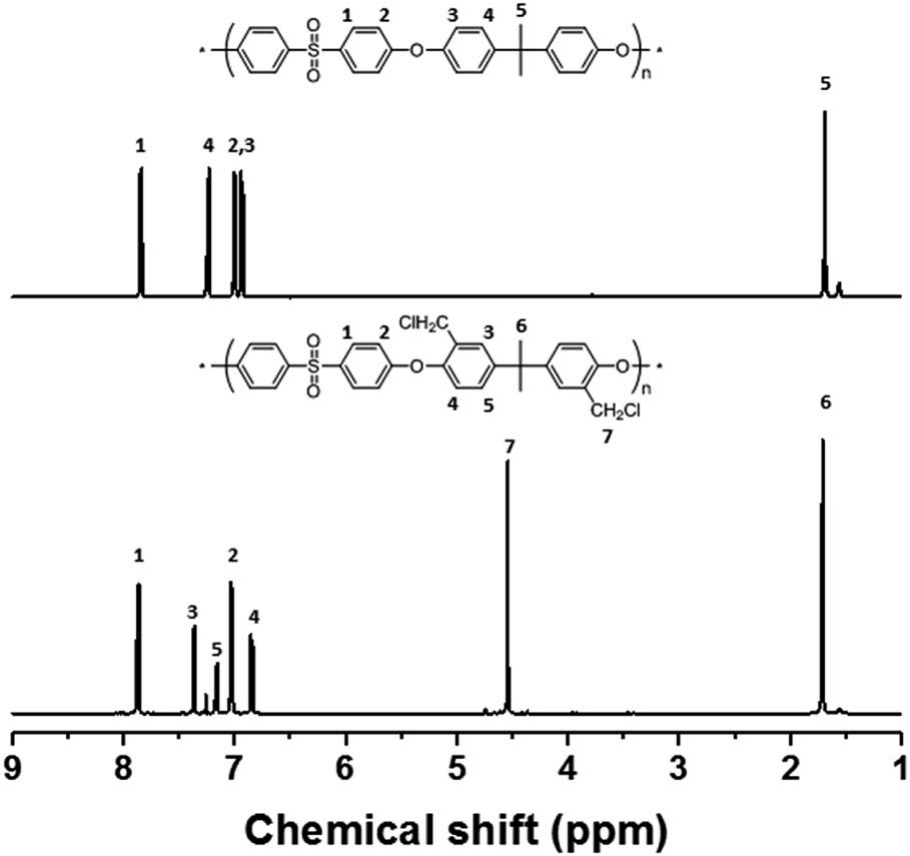
^1^H NMR spectra of poly(arylene ether sulfone) random copolymer before and after chloromethylation.

It appears to be impossible to prepare a fully functionalized PAES multi-block copolymer, with 100% DC, without side reactions, irrespective of the reaction conditions. For comparison, chloromethylation was also carried out using a PSU random copolymer (R–C-1) following the procedure used for the PAES multi-block copolymer. Fig. 4 shows ^1^H NMR spectra of the PSU random copolymer before and after chloromethylation, which reveals full conversion. A DC of 100% (two chloromethyl groups on each biphenyl unit) was observed even after a short reaction time (2 h). In addition, the product was very soluble in CDCl_3_ at room temperature, and the NMR sample was easily filtered through a 0.2 μm PTFE syringe filter. Consequently, the chloromethylation of a multi-block copolymer appears to be accompanied by side reactions (Friedel–Crafts alkylation) at DCs over 80%, whereas a random copolymer is almost fully converted without any observable crosslinking. Similar attempts to functionalize the multi-block copolymer through Friedel–Crafts acylation with 6-bromohexanoyl chloride gave a material that was partially insoluble owing to crosslinking after functionalization; the filtered soluble component was unreacted multi-block copolymer as evidenced by the lack of any change in the ^1^H NMR spectrum. On the other hand, the random copolymer was fully functionalized with bromoacyl groups (see Scheme S2, Fig. S4 and S5†).

These observations may be the result of the proximity between active sites in the multi-block architecture, while the active sites are randomly distributed in the random copolymer. When one of the active sites is functionalized, an adjacent active site is sterically restricted and is available for the further reaction. In this respect, functional groups in the random copolymer are sparsely introduced at active sites during the initial stages, with the remaining active sites randomly participating in further functionalization. On the other hand, each hydrophilic block in the multi-block architecture is composed of a few or few tens of repeating units. The active sites in these hydrophilic blocks are very close to each other; consequently, these clustered active sites have an increased chance of reacting among themselves rather than undergoing the desired chloromethylation reaction. Consequently, self-reaction (Friedel–Crafts crosslinking) of the active sites in the block copolymer occurs more frequently compared to those in a random copolymer.

### Bromination

**Fig. 5:**
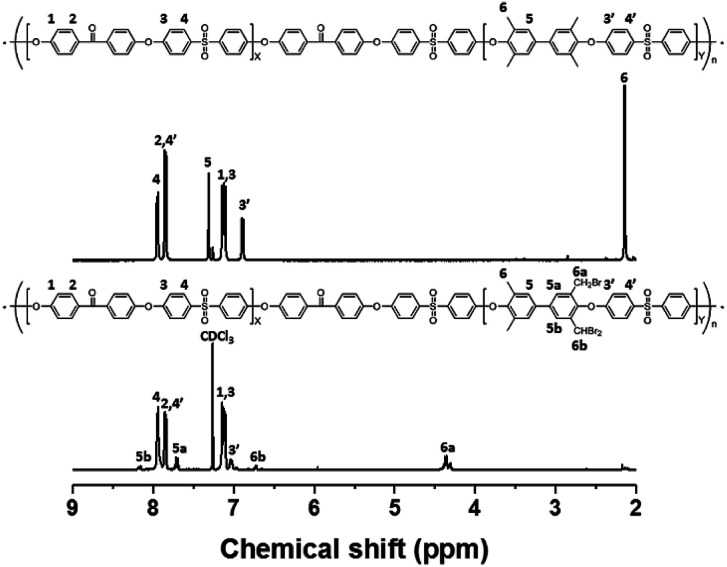
^1^H NMR spectra of a poly(arylene ether sulfone) multi-block copolymer before and after bromination.

**Scheme 3:**
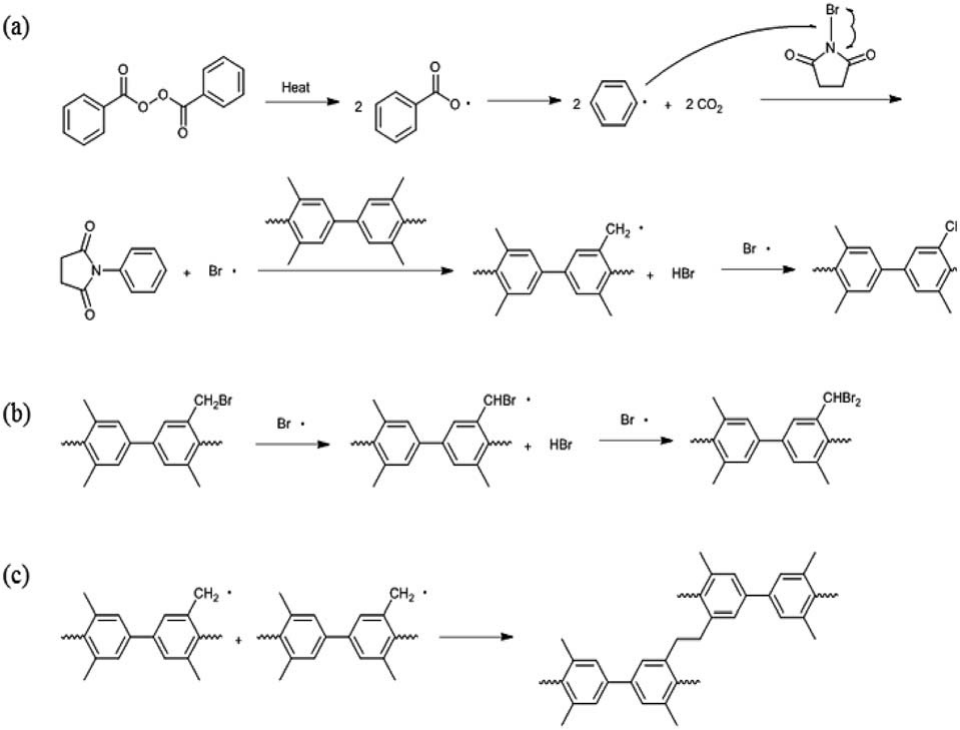
Potential mechanisms for (a) bromination, (b) di-bromination, and (c) crosslinking.

Bromination followed by quaternization of the formed benzylic bromides is also a method for the post-functionalization of multi-block copolymers for AEM applications. For bromination experiments, the multi-block-copolymer precursor was synthesized from a ketone-containing hydrophobic oligomer and a tetramethyl-substituted biphenyl-containing hydrophilic oligomer, and was characterized by ^1^H NMR spectroscopy and GPC (Scheme S3, Fig. S6 and S7†). The tetramethyl-substituted biphenyl unit was chosen as it provides selective active sites for radical bromination. The general synthetic procedure is shown in Scheme 1b. BPO or AIBN was used as the radical initiator, while NBS was used as the brominating reagent. Chemical structures before and after bromination were identified by ^1^H NMR spectroscopy, as shown in Fig. 5. The peak corresponding to the benzylic methyl groups (proton 6 at 2.1 ppm) disappeared and a new peak at 4.3 ppm appeared following bromination. Moreover, peaks corresponding to protons 3′ and 5a (Fig. 5) were slightly shifted downfield. Based on this NMR evidence, we conclude that the methyl groups were converted into bromomethyl groups. However, some small unexpected peaks (protons 5b at 8.1 ppm and 6b at 6.7 ppm) were observed in the ^1^H NMR spectrum of the mono-brominated multi-block copolymer (Fig. S8†) following bromination, which are assigned to the protons of di-bromomethyl units. The degree of mono-bromination (DB) was calculated by integrating the signals for protons 6a and 4, while the degree of di-bromination (DDB) was determined from the peaks for protons 6b and 4; DB and DDB values of 72% and 15% were obtained, respectively. Radical bromination can lead to over-bromination (di-bromination) and can be also accompanied by several side reactions that include crosslinking, as shown in Scheme 3.^17^ These byproducts hinder further functionalization, such as quaternization for AEM applications, and can lead to degradation and poor solubility. Therefore, we conclude that the bromination reaction needs to be carefully controlled in order to obtain a high DB and diminished di-bromination and crosslinking.

**Fig. 6:**
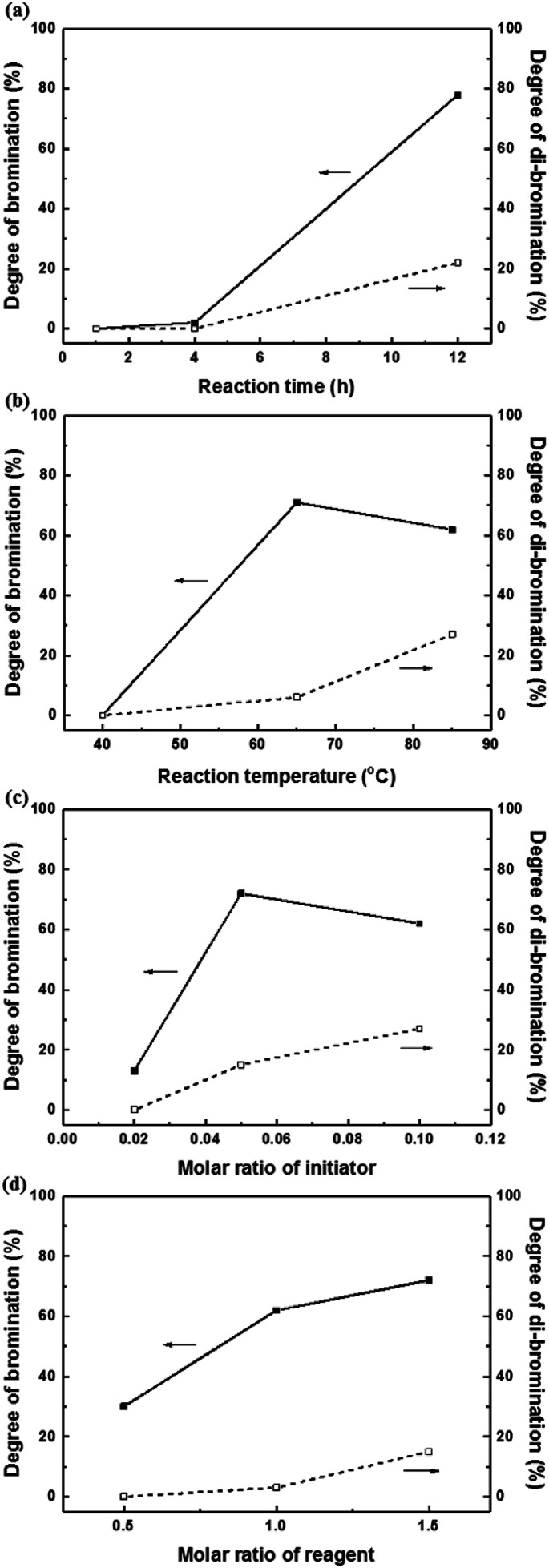
The degrees of bromination (solid lines) and di-bromination (broken lines) as functions of (a) reaction time, (b) reaction temperature, (c) the molar ratio of initiator, and (d) the molar ratio of reagent.

Harsh reaction conditions (high concentrations of radicals or high temperatures) and prolonged reaction times may cause significant side-reactions to occur, as discussed. Radical-initiated bromination can be affected by the reaction time, the thermal-initiation temperature, the ratios of the reagent and initiator to the active sites; consequently, an exact understanding of the bromination process is required in order to control the reaction. Hence, we varied four important factors (reaction time and temperature, and the amounts of initiator and NBS) in order to optimize the reaction and better understand the radical-initiated bromination mechanism. A series of brominated PAES-based polymers (B–B-*x*, where “B–B” refers to brominated block copolymer, and *x* is the batch number) were prepared to order to determine the optimized bromination conditions, the results of which are summarized in Table 2. BPO and NBS were selected as the thermal initiator and brominating reagent, respectively. We first studied the effect of reaction time on DB and DDB at 65 °C with 0.05 equiv. of BPO and 2.0 equiv. of NBS. Although a DB of 78% was observed after 12 h, a high DDB of 22% was also observed, and almost no bromination occurred during the first 4 h, as shown in Fig. 6a. Moreover, the product was partially insoluble, which is ascribable to crosslinking. We conclude that excess reagent generates too many bromine radicals, and that long reaction times result in crosslinking through termination or radical bromination. In addition, the reaction temperature and the amount of initiator were not appropriate for initiating this reaction. For these reasons, the reaction temperature was varied between 40 and 85 °C and 0.10 equiv. of BPO and 1.5 equiv. of NBS were used, with the reaction time fixed at 3 h; longer reaction times increase the probability of undesirable side reactions, such as di-bromination and crosslinking, because no radical-terminating reagents are present in the reaction mixture. Fig. 6b displays DB and DDB values as functions of reaction temperature. A short reaction time (3 h) was sufficient to ensure bromination at 65 °C, whereas 40 °C was insufficient to thermally initiate the radical reaction with BPO, resulting in no bromination. Nonetheless, the total conversion of methyl groups into the corresponding bromomethyl and di-bromomethyl groups was less than 80% at 65 °C. Clearly, 85 °C provides a better outcome than 65 °C, but the conditions require further optimization to reduce the DDB. Fig. 6c and d show the results of samples B–B-6 to B–B-10 with varying amounts of initiator and reagent. When the amount of initiator was reduced from 0.10 to 0.05 equiv., the DB was observed to increase, while the DDB decreased. Milder conditions are better than harsher ones, since fast initiation forms excess radicals that promote a high DDB. However, 0.02 equiv. of initiator or less than 1.0 equiv. of reagent did not provide high DBs.

**Table 2:** Summary of the bromination reaction conditions

Sample	Initiator^a^ (equiv.)	Reagent^b^ (equiv.)	Temperature (°C)	Time (h)	DB (%)	DDB (%)
B–B-1	0.05	2.0	65	1	0	0
B–B-2	0.05	2.0	65	4	2	0
B–B-3	0.05	2.0	65	12	78	22
B–B-4	0.10	1.5	40	3	0	0
B–B-5	0.10	1.5	65	3	71	6
B–B-6	0.10	1.5	85	3	62	27
B–B-7	0.05	1.5	85	3	72	15
B–B-8	0.02	1.5	85	3	13	0
B–B-9	0.05	1.0	85	3	62	3
B–B-10	0.05	0.5	85	3	30	0
B–B-11	0.05	1.5	85	3	76	12
B–B-12	0.10	1.5	85	3	74	17

a Molar ratio of initiator to active sites (B–B-1 to B–B-10, initiator = BPO; B–B-11 to B–B-12, initiator = AIBN).

b Molar ratio of reagent to active sites.

We adjusted the amounts of initiator and reagent, the reaction temperature, and the time, using BPO the initiator, in attempts to obtain a DB of 100% without any side reactions. In addition, AIBN was explored as the initiator to examine the effect of the type of initiator on the bromination reaction. Despite these efforts, similar DB and DDB values to those obtained using BPO as initiator were observed, as shown in Fig. S9.† Di-bromination was always observed when DB exceeded 60%, irrespective of the reaction conditions. We attribute this to clustered bromination sites on the hydrophilic blocks, as was observed during the chloromethylation experiments. Consequently, we conclude that post-functionalization through chloromethylation or bromination of a multi-block copolymer is not a good approach to a fully functionalized polymer that maintains a block-copolymer architecture.

### A tetra-trimethylbenzylammonium-bearing multi-block copolymer (B-Q-1)

**Scheme 4:**
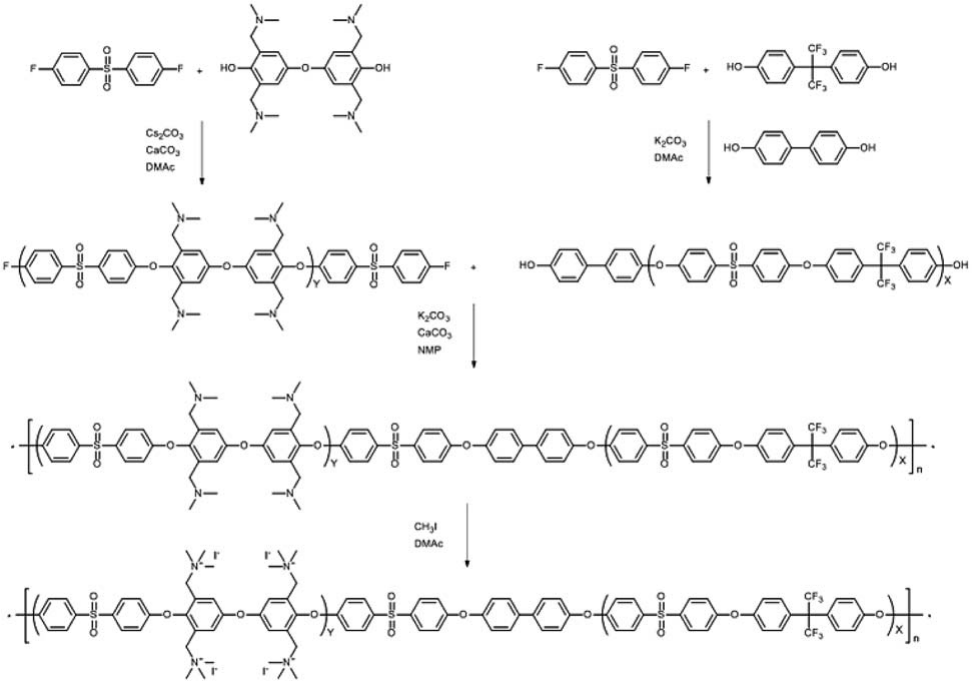
Synthesis of the tetra-(trimethylbenzylammonium)-functionalized multi-block copolymer.

An alternative way of preparing a fully functionalized multi-block copolymer is shown in Scheme 4. Hygroscopic ionic groups easily absorb water molecules that deactivate the base catalyst during the condensation reaction. For this reason, a diarylether unit bearing four dimethylamino groups was selected because amino groups can be quarternized through methylation in the absence of side reactions.^28^ The since the amine-containing hydrophilic oligomer was unstable above 140 °C, it was synthesized at 140 °C. Consequently, Cs_2_CO_3_ was used instead of K_2_CO_3_ as the catalyst in order to enhance reactivity at the low temperature; CaCO_3_ also inhibits ether–ether exchange during oligomerization.^14,27^ The hydroxyl-group-terminated hydrophobic oligomer was prepared in a two-step process involving the oligomerization of DFDPS with 6F-BPA, followed by end-capping with BP. The chemical structures of the hydrophilic and hydrophobic oligomers were confirmed by ^1^H NMR spectroscopy (Fig. S10†), which revealed peaks corresponding to the end groups as well as protons in the main repeating units, verifying that the halide-terminated hydrophilic oligomer and the hydroxyl-terminated hydrophobic oligomer had been successfully prepared.

**Fig. 7:**
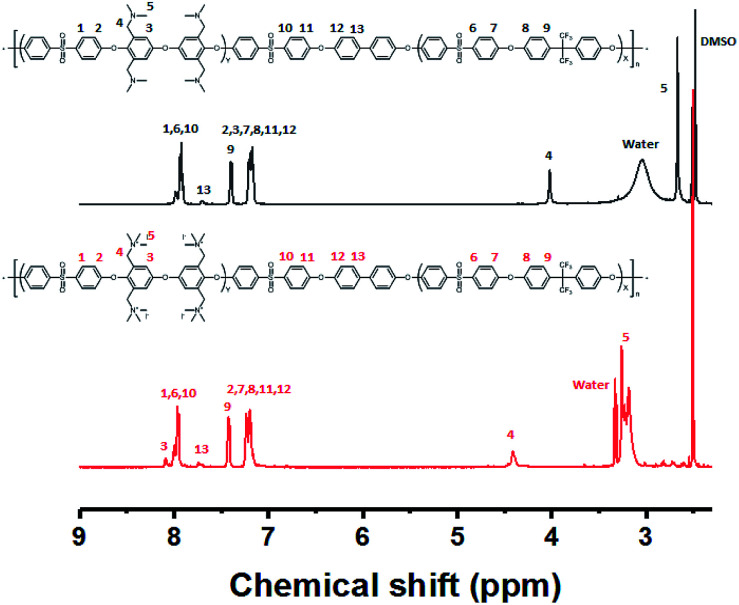
^1^H NMR spectrum of the B-Q-1 before and after quaternization.

The multi-block copolymer was synthesized by the coupling of the hydrophilic and hydrophobic oligomers using a similar method to that used for oligomerization. The multi-block copolymer had a high molecular weight (98 kDa) (Fig. S11†), indicating a lack of ether–ether exchange reactions. In addition, the multi-block copolymer showed clear ^1^H NMR spectra before and after quaternization (Fig. 7). The sharp peaks the protons 3, 4, and 5 indicate a fully functionalized chemical structure, whereas the ^1^H NMR spectra of the chloromethylated multi-block copolymers always showed a peak from unreacted material (proton 5 in Fig. 1) and the brominated multi-block copolymers had peaks corresponding to the undesired di-brominated moieties (protons 5b and 6b in Fig. 5) indicating loss of block copolymer architecture. Moreover, the significant shift in the peaks of protons 3, 4, and 5 indicated successful quaternization. The calculated IEC value of B-Q-1 membrane, 1.6 meq g^−1^, was lower than that of commercial FAA-3-30 (1.9–2.1 meq g^−1^).

**Fig. 8:**
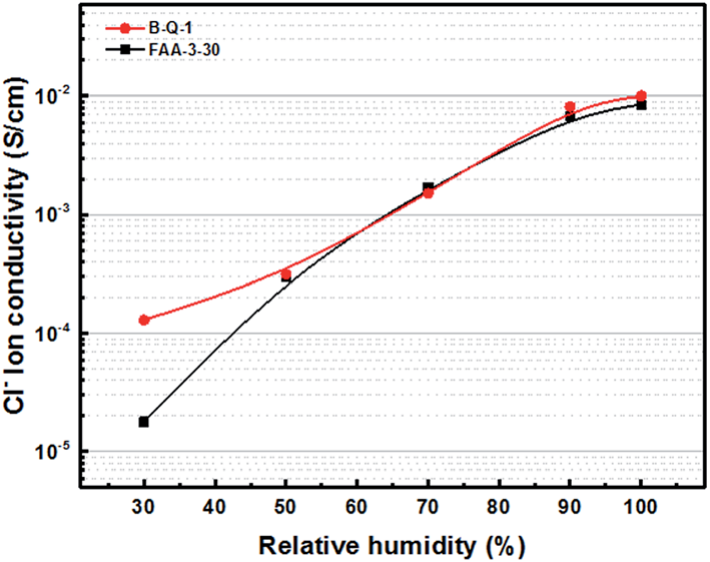
Chloride anion conductivity of B-Q-1 and FAA-3-30 membranes at 70 °C.

**Fig. 9:**
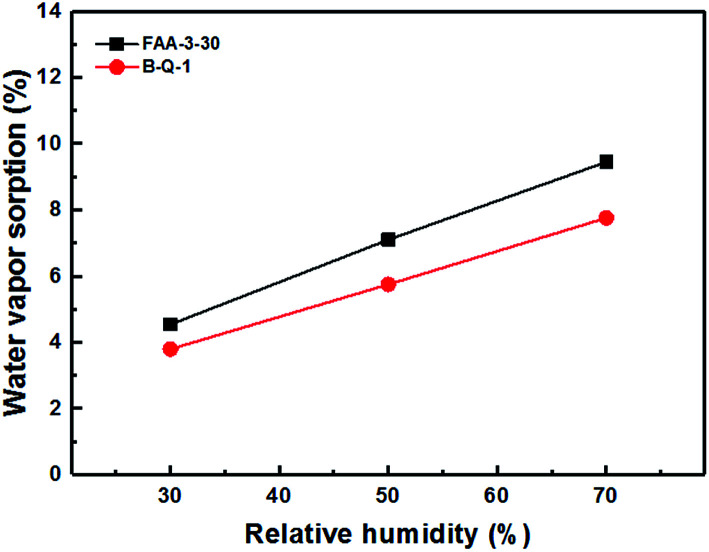
Water vapor sorption of B-Q-1 and FAA-3-30 membranes at 70 °C.

The B-Q-1 multi-block copolymer had good solubility in DMAc and the polymer solution penetrated easily through a 0.45 μm syringe filter, supporting the absence of crosslinking. It is well-known that hydroxide anion has the highest ion conductivity among various anions such as chloride, bromide, and so on. However, it is easily poisoned by carbon dioxide leading to the formation of carbonate species which results in decreased ion conductivity.^29^ Due to these reasons, the B-Q-1 and the commercial FAA-3-30 membranes were treated with aqueous 1 M NaCl solution to diminish the effect of counterion of the cationic functional groups. Although B-Q-1 and FAA-3-30 membranes showed similar chloride anion conductivity at high RH conditions (>70% RH), the ion conductivity of B-Q-1 was less dependent on RH (Fig. 8). Moreover, the water uptake of B-Q-1 (13%) was lower than that of FAA-3-30 (15%) at room temperature. Similarly, the B-Q-1 membrane showed lower water vapor sorption than the FAA-3-30 membrane at low RH (≤70%), as shown in Fig. 9. The relatively higher conductivity of B-Q-1 even at the lower level of water uptake might be one of the indicators for the successful synthesis of the block copolymer architecture with densely concentrated ammonium groups.

The alkaline membrane fuel cells developed until now are suitable for operation at a fully hydrated condition due to their low ion conductivity and poor chemical stability at low humidity or dry conditions.^9,29,30^ However, further development is necessary to operate it under ambient conditions for broader and real-world applications. In this respect, our synthetic approach for preparation of authentic multi-block copolymers with high ion conductivity under low RH conditions is a very attractive method for developing advanced AEMs. We are currently expanding this synthetic technique to various chemical structures to prepare multi-block AEMs of improved performance.

## Conclusions

Poly(arylene ether sulfone) multi-block copolymers, as precursors for post-functionalization by chloromethylation or bromination, were successfully synthesized by nucleophilic aromatic substitution. Chloromethyl or bromomethyl functional groups were selectively introduced into the hydrophilic blocks since electron-withdrawing groups (*e.g.*, sulfone and ketone) deactivate the hydrophobic blocks toward electrophilic aromatic substitution, while benzylic methyl substituents were the only active sites available for radical bromination. Reaction conditions were optimized by varying the amounts of catalyst and reagent, as well as the reaction times and temperatures; however, undesired side reactions were always observed during chloromethylation and bromination. Maximum values of DC and DB were around 80 and 70%, respectively, and insoluble components were obtained when the degrees of functionalization were above these values, indicative of the formation of crosslinked by-products. In particular, di-bromination was also observed during the bromination process, which results in degradation and interferes with further quaternization reactions. Although the multi-block copolymers failed to be fully functionalized, random copolymers were fully converted without any side reactions. We ascribe this observation to clustered active sites in the multi-block copolymers that increase the chances of reactions among themselves rather than with the desired reagents. We conclude that the introduction of functional groups into monomers prior to multi-block polymerization is the most effective method for the synthesis of multi-block copolymers for AEM applications. The resulting multi-block copolymer, synthesized from functionalized monomer, showed superior anion conductivity than the commercial membrane even under low RH conditions, showcasing the potential of the current study in setting the course for development of advanced AEMs based on multi-block copolymers.

## Conflicts of interest

There are no conflicts to declare.

## Supplementary Material

RA-009-C9RA03888D-s001
