# Supplementary material for: Synthetic approaches for advanced multi-block anion exchange membranes
Source: RSC Adv. 2019 Jul 5;9(37):21106–15. doi: 10.1039/c9ra03888d (PMC9065993; doi:10.1039/c9ra03888d)
Supplement: RA-009-C9RA03888D-s001 [file RA-009-C9RA03888D-s001.pdf]

Electronic Supplementary Material (ESI) for RSC Advances.
This journal is © The Royal Society of Chemistry 2019

Supporting Information

Synthetic approaches for advanced multi-block anion exchange membranes

Dongwon Shin,[a] *Adam F. Nugraha,*[ab] *Farid Wijaya,*[ab] *Sojeong Lee,*[a] *Eunyoung Kim,*[a] *Jieun Choi,*[c] *Hyoung-Juhn Kim,*[c] *and Byungchan Bae**[ab]

*E-mail: bcbae@kier.re.kr

[a]Fuel Cell Laboratory, Korea Institute of Energy Research, Daejeon 34129, Republic of Korea

[b]Department of Renewable Energy Engineering, University of Science and Technology, Daejeon 34113, Republic of Korea

[c]Fuel Cell Research Center, Korea Institute of Science and Technology, Seoul 02792, Republic of Korea

EXPERIMENTAL

Materials. Bis(4-fluorophenyl) sulfone (DFDPS), 4,4'-dihydroxybenzophenone (DHBP), 4,4'-dihydroxybiphenyl (BP), 4,4'-(hexafluoroisopropylidene)diphenol (6F-BPA), 3,3',5,5'-tetramethylbiphenyl-4,4'-diol (TMBP), K_2CO_3, $CaCO_3$, $AlCl_3$, 6-bromohexanoyl chloride (BHC), 1,1,2,2-tetrachloroethane (TCE), *N,N*-dimethylacetamide (DMAc), and deuterated *N,N*-dimethyl sulfoxide (DMSO-d_6) were purchased from Sigma-Aldrich.

Synthesis of multi-block copolymers for chloromethylation and bromination studies. The same hydroxyl-group-terminated oligomer was used as the hydrophobic-oligomer precursor to the multi-block copolymers used in both chloromethylation and bromination studies; it was synthesized by the nucleophilic aromatic substitution reaction between DHBP and DFDPS. DHBP (9.34 mmol, 2.00 g) and DFDPS (8.40 mmol, 2.14 g) were dissolved in DMAc (18.7 mL) and the temperature was slowly raised to 140 °C. After adding K_2CO_3 (18.67 mmol, 2.58 g) and $CaCO_3$ (93.36 mmol, 9.35 g), oligomerization was performed at 140 °C for 4 h. The oligomer was collected by filtration following precipitation of the solution into 1 M aqueous HCl, after which it was washed with methanol to remove impurities, and dried at 80 °C under vacuum.

The oligomer for the hydrophilic block of the polymer used in the chloromethylation studies was synthesized using a fluorine-terminated end-group to facilitate coupling with the hydroxyl-terminated hydrophobic oligomer. DFDPS (11.82 mmol, 3.00 g), BP (10.74 mmol, 2.00 g), DMAc (21.5 mL) were placed in a flask and the temperature increased to 130 °C. After adding K_2CO_3 (12.89 mmol, 1.78 g), oligomerization was performed for 4 h. The reaction solution was added dropwise into a large excess of D.I. water and methanol for purification. The resulting product was collected by filtration and dried under vacuum.

The reactive component of the precursor polymer for the bromination studies was prepared by dissolving TMBP (16.50 mmol, 4.00 g) and DFDPS (18.60 mmol, 4.00 g) in DMAc (30.0 mL) at 140 °C. The reaction was activated by the addition of K_2CO_3 (33.00 mmol, 4.60 g) as the catalyst. After 4 h, the mixture was poured into excess D.I. water and methanol for purification. The crude product was collected by filtration and dried at 80 °C under vacuum.

The precursor multi-block copolymers were synthesized by coupling the hydrophobic and hydrophilic oligomers in a 1:1 molar feed ratio, with 2 equiv. of K_2CO_3 added as a catalyst and 5 equiv. of $CaCO_3$ added to inhibit ether-ether exchange reactions. Polymerization was conducted at 140 °C for 8 h. The final product was precipitated into 1 M aqueous HCl and washed several times with methanol. The white fibrous polymer was filtered and dried under vacuum.

Friedel–Crafts acylation. The precursor multi-block copolymer (2.21 mmol, 0.50 g) was dissolved in TCE (20 mL). The solution was cooled to 0 °C and $AlCl_3$ (4.41 mmol, 0.59 g) and BHC (4.41 mmol, 0.94 g) were slowly added to the polymer solution. The temperature was increased to 40 °C and the mixture was stirred for 3 h. After cooling to room temperature, the solution was precipitated into ethanol. The product collected by filtration, washed several times with ethanol and dried under vacuum. The same procedure was followed for the polysulfone random copolymer.

SCHEMES AND FIGURES

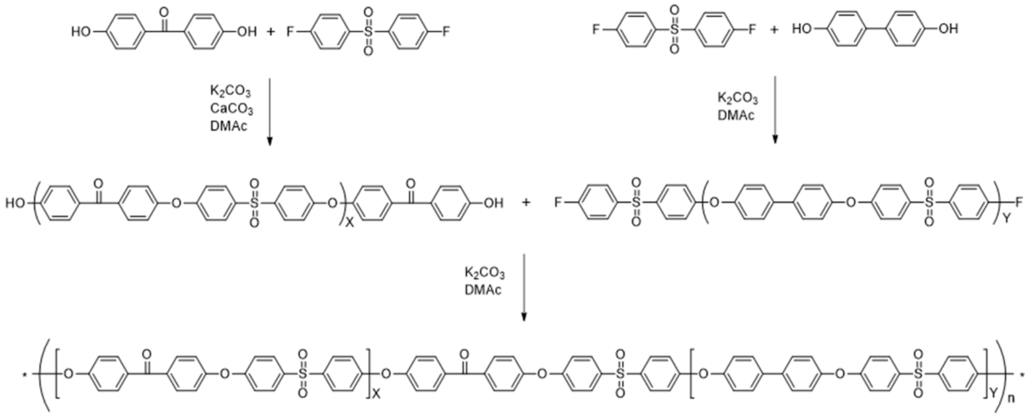

Scheme S1. Synthesis of the multi-block copolymer for chloromethylation studies.

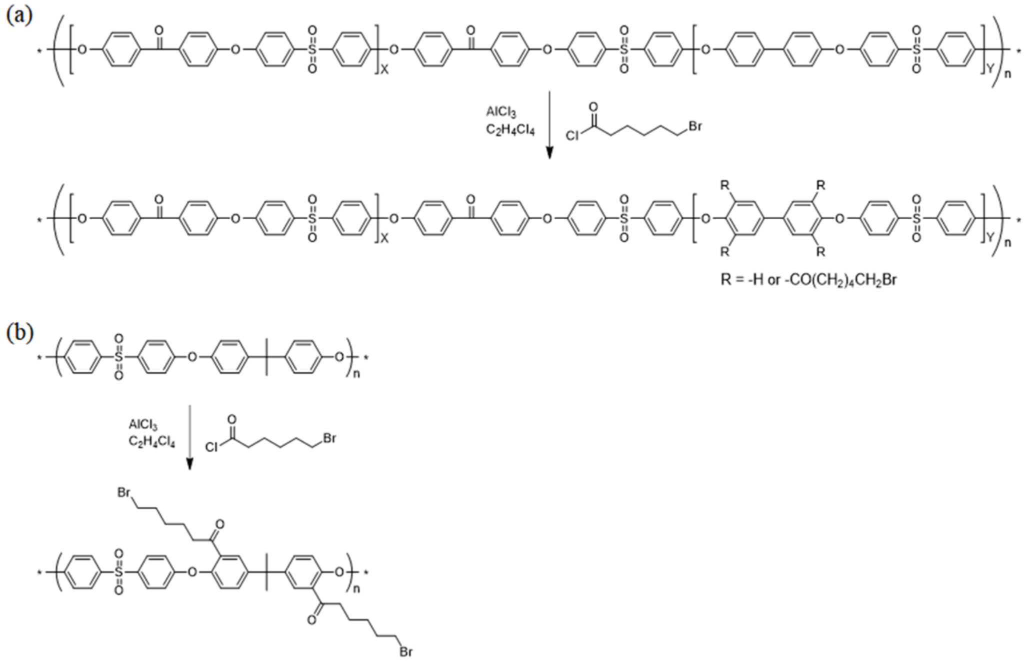

Scheme S2. Friedel–Craft acylation of (a) the multi-block copolymer and (b) the random copolymer.

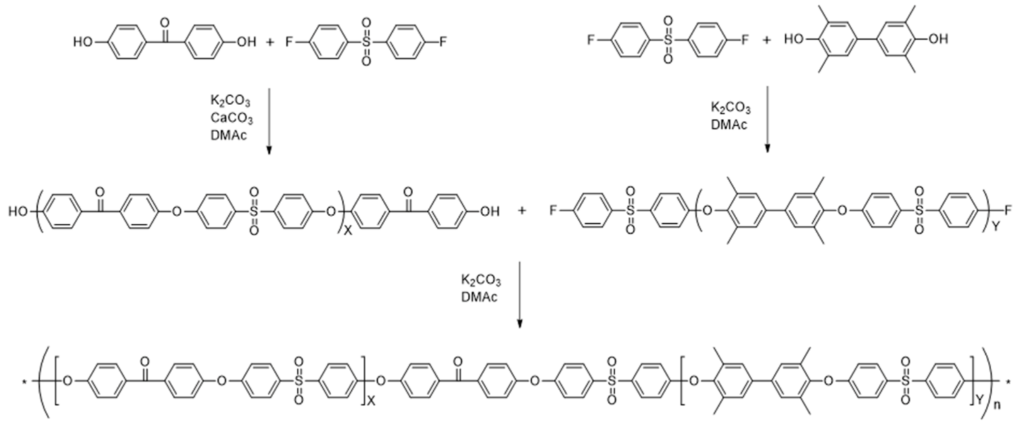

Scheme S3. Synthesis of the multi-block copolymer used in bromination studies.

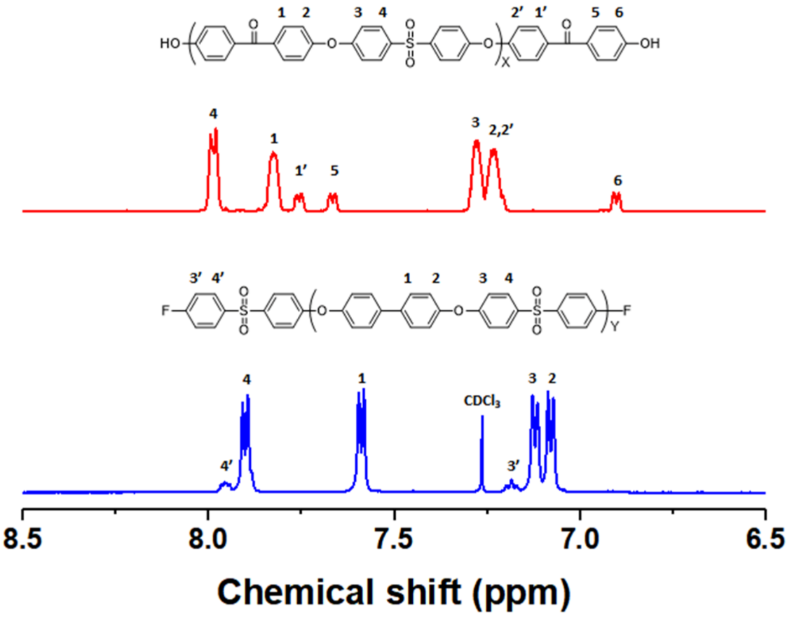

Figure S1. ^{1}H NMR spectra of oligomers for the precursor multi-block copolymer used in chloromethylation studies.

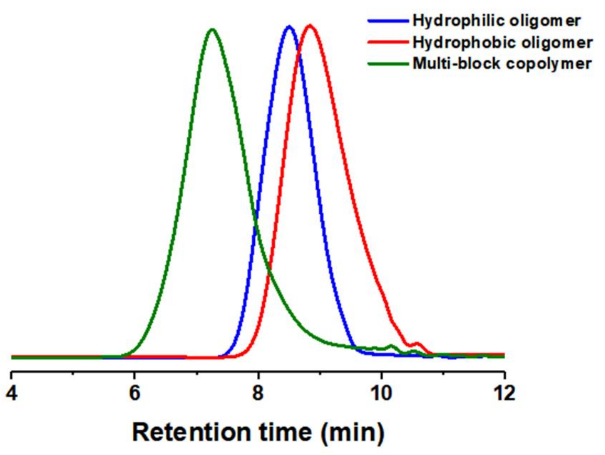

Figure S2. GPC traces of the hydrophilic and hydrophobic oligomers, and the multi-block copolymer precursor used in chloromethylation studies.

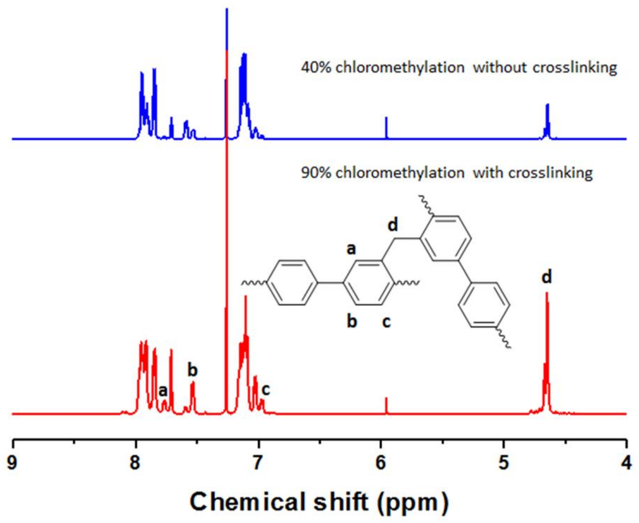

Figure S3. ^{1}H NMR spectra of crosslinked poly(arylene ether sulfone) multi-block copolymers after chloromethylation.

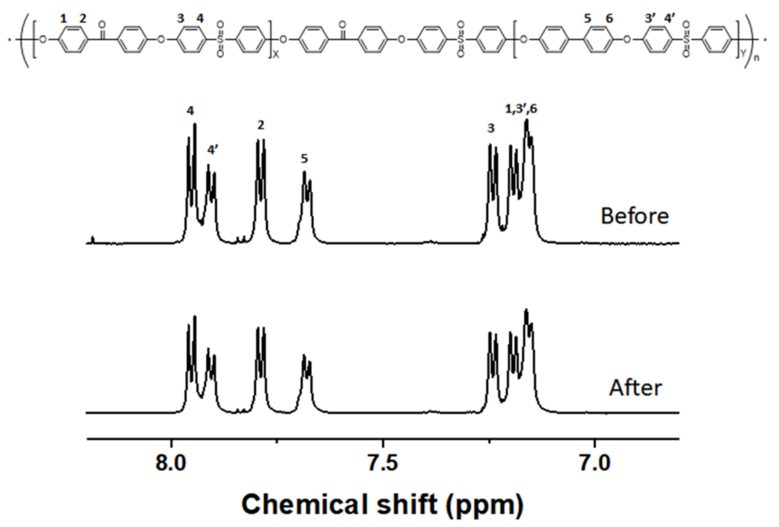

Figure S4. ^{1}H NMR spectra of the poly(arylene ether sulfone) multi-block copolymer before and after acylation.

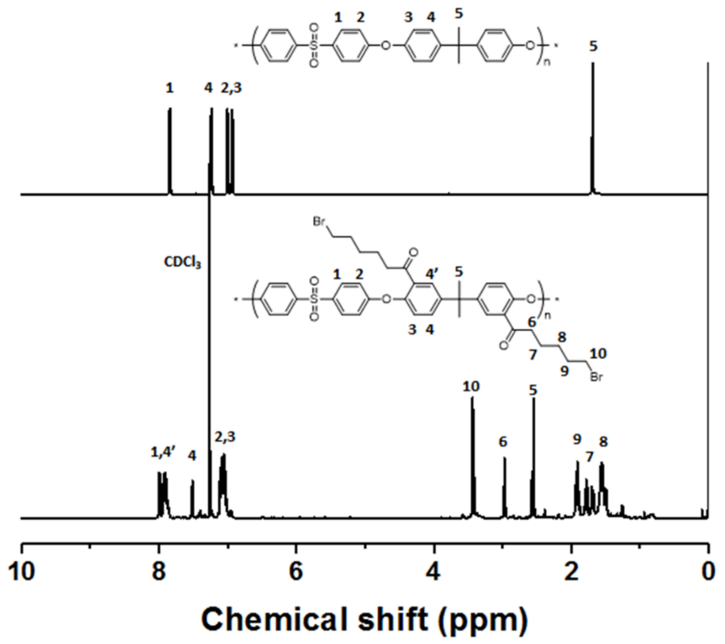

Figure S5. ^{1}H NMR spectra of the poly(arylene ether sulfone) random copolymer before and after acylation.

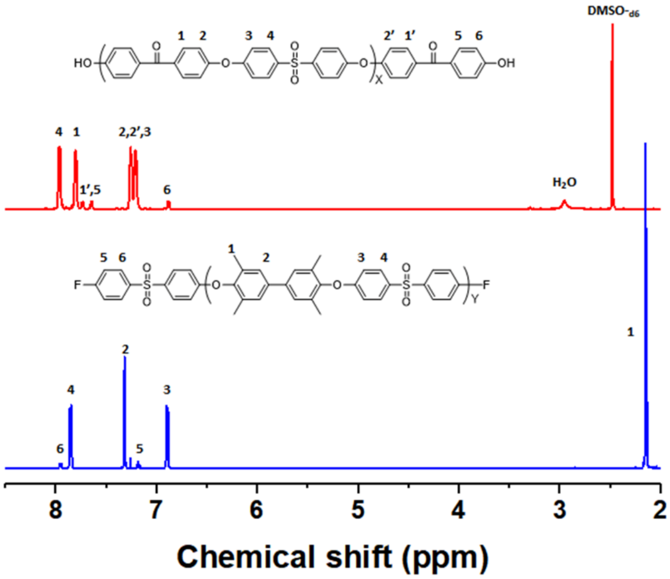

Figure S6. ^{1}H NMR spectra of oligomers for the precursor multi-block copolymer used in bromination studies.

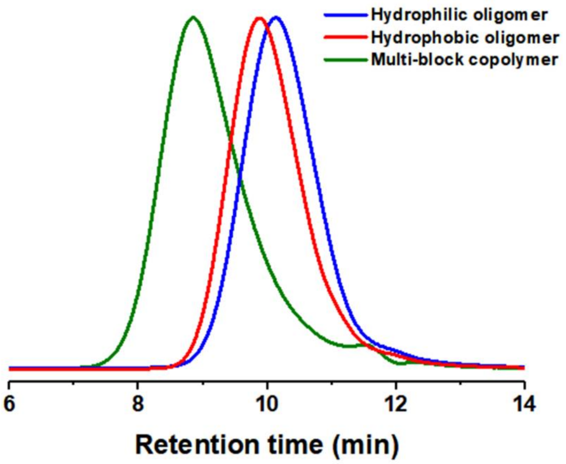

Figure S7. GPC traces of the hydrophilic and hydrophobic oligomers, and the multi-block copolymer precursor used in bromination studies.

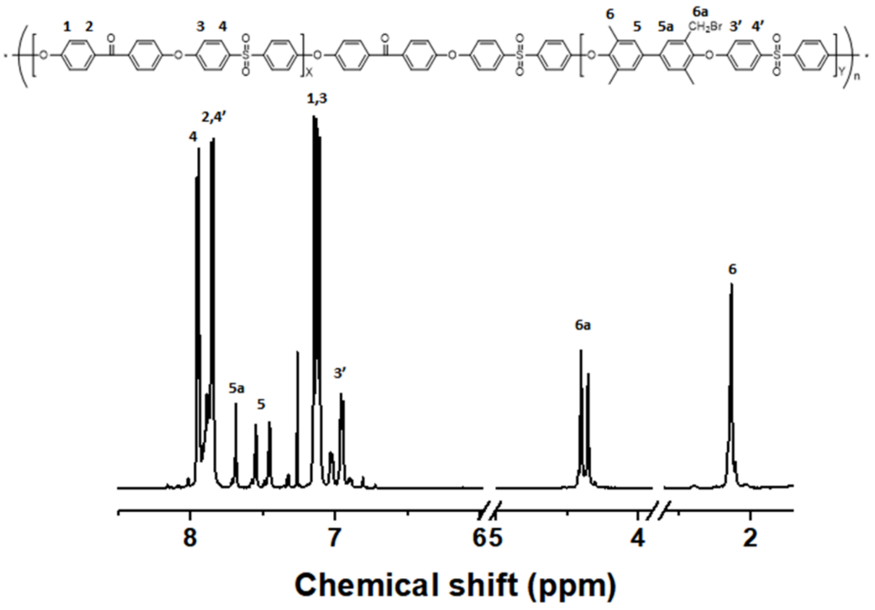

Figure S8. ^{1}H NMR spectrum of the mono-brominated poly(arylene ether sulfone) multi-block copolymer.

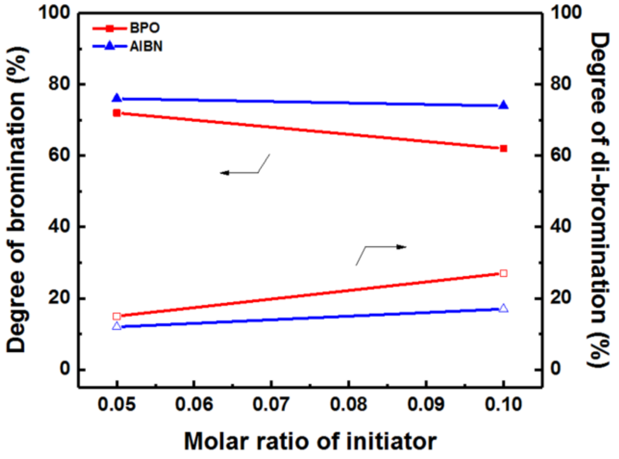

Figure S9. Degree of bromination (solid symbols) and dibromination (open symbols) as functions of molar ratio of initiator.

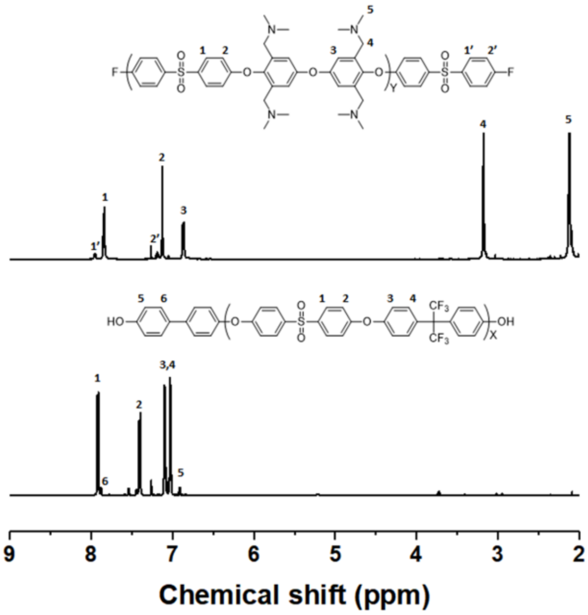

Figure S10. ^{1}H NMR spectra of the hydrophilic and hydrophobic oligomers.

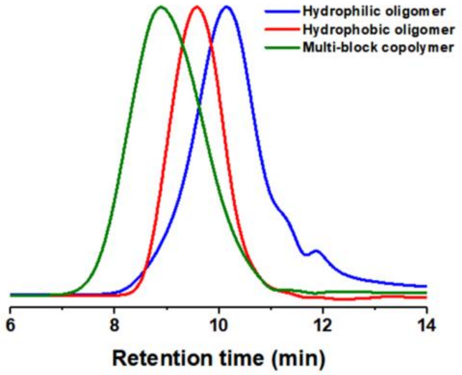

Figure S11. GPC traces of the hydrophilic and hydrophobic oligomers, and the multi-block copolymer.
